# Serial Histopathological Examination of the Lungs of Mice Infected with Influenza A Virus PR8 Strain

**DOI:** 10.1371/journal.pone.0021207

**Published:** 2011-06-20

**Authors:** Masaya Fukushi, Tateki Ito, Teruaki Oka, Toshio Kitazawa, Tohru Miyoshi-Akiyama, Teruo Kirikae, Makoto Yamashita, Koichiro Kudo

**Affiliations:** 1 Disease Control and Prevention Center, National Center for Global Health and Medicine, Tokyo, Japan; 2 Department of Infectious Diseases, Research Institute, National Center for Global Health and Medicine, Tokyo, Japan; 3 Department of Diagnostic Pathology, National Center for Global Health and Medicine, Tokyo, Japan; 4 Department of Pathology, Kanto Central Hospital, Tokyo, Japan; 5 Biological Research Laboratories, Daiichi Sankyo Co. Ltd., Tokyo, Japan; Erasmus Medical Center, The Netherlands

## Abstract

Avian influenza H5N1 and pandemic (H1N1) 2009 viruses are known to induce viral pneumonia and subsequent acute respiratory distress syndrome (ARDS) with diffuse alveolar damage (DAD). The mortality rate of ARDS/DAD is extremely high, at approximately 60%, and no effective treatment for ARDS/DAD has been established. We examined serial pathological changes in the lungs of mice infected with influenza virus to determine the progress from viral pneumonia to ARDS/DAD. Mice were intranasally infected with influenza A/Puerto Rico/8/34 (PR8) virus, and their lungs were examined both macro- and micro-pathologically every 2 days. We also evaluated general condition, survival rate, body weight, viral loads in lung, and surfactant proteins in serum. As a result, all infected mice died within 9 days postinfection. At 2 days postinfection, inflammation in alveolar septa, i.e., interstitial pneumonia, was observed around bronchioles. From 4 to 6 days postinfection, interstitial pneumonia with alveolar collapse expanded throughout the lungs. From 6 to 9 days postinfection, DAD with severe alveolar collapse was observed in the lungs of all of dying and dead mice. In contrast, DAD was not observed in the live infected-mice from 2 to 6 days postinfection, despite their poor general condition. In addition, histopathological analysis was performed in mice infected with a dose of PR8 virus which was 50% of the lethal dose for mice in the 20-day observation period. DAD with alveolar collapse was observed in all dead mice. However, in the surviving mice, instead of DAD, glandular metaplasia was broadly observed in their lungs. The present study indicates that DAD with severe alveolar collapse is associated with death in this mouse infection model of influenza virus. Inhibition of the development of DAD with alveolar collapse may decrease the mortality rate in severe viral pneumonia caused by influenza virus infection.

## Introduction

Pandemic (H1N1) 2009 influenza A virus has spread worldwide since 2009. Many people have been infected with this new influenza virus, some of whom became seriously ill and required respiratory care [1]–[4]. Avian H5N1 virus infection is also known to have a high mortality rate. The main cause of death among patients with viral pneumonia caused by pandemic (H1N1) 2009 and avian H5N1 influenza virus is acute respiratory distress syndrome (ARDS) [2], [4]–[10], which is clinically defined as acute respiratory failure, bilateral infiltration in chest X-rays, low oxygen in arterial blood, and normal cardiac filling pressure [11]–[13]. ARDS is caused by several etiologies, including viral or bacterial infection in the lung and sepsis. However, autopsies of patients with ARDS have found a pathologically identical characteristic, called diffuse alveolar damage (DAD), which is defined by the formation of a hyaline membrane lining the alveoli and alveolar ducts, inflammatory cell accumulation in the lungs, and pulmonary edema [14]–[16]. Although effective anti-influenza virus drugs are currently available, the mortality rate of ARDS caused by influenza virus remains high. Therefore, it is necessary to deepen our understanding of ARDS/DAD in order to develop an effective treatment.

Viral pneumonia and subsequent ARDS caused by influenza virus has been investigated in mice [17]–[21]. In addition, DAD in mice has only recently been reported [22]. Therefore, to understand the histopathological process from viral pneumonia to DAD, we performed serial pathological analysis of lungs from mice infected with mouse-adapted influenza A/Puerto Rico/8/34 (PR8, H1N1) virus, which was lethal to mice. The results demonstrated that death in infected mice was closely associated with expansion of DAD in the lungs. Our results suggest that inhibition of the development of DAD in the lungs through medical intervention may decrease the mortality rate of viral pneumonia and subsequent ARDS caused by influenza virus infection.

## Results

### General Appearance, Survival Rate, Body Weight and Viral Load

**Figure 1:**
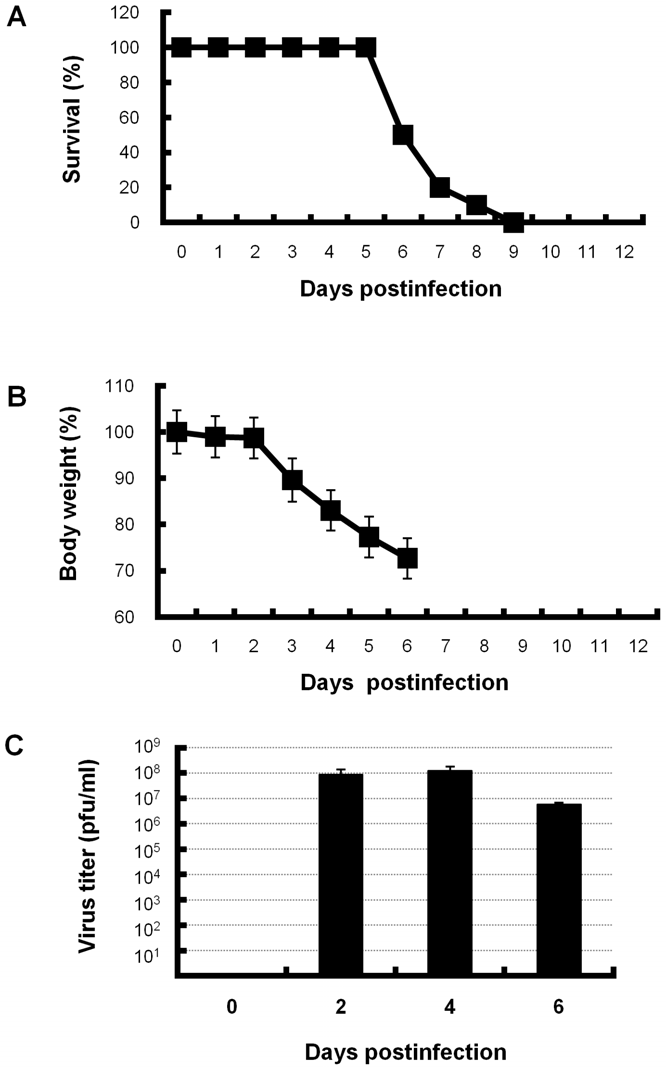
Survival rate, body weight and viral titers in lungs of PR8-infected mice. Mice were intranasally inoculated with PR8 virus. (A) Survival rates of PR8-infected mice (n = 10). (B) Body weight of PR8-infected mice (0–5 days postinfection, n = 10; 6 days postinfection, n = 5). Percentages of mean body weight ± SD based on day 0 are shown. (C) Viral titers in lungs of PR8-infected mice (n = 5, each day) was measured by plaque forming assay using MDCK cells at 2, 4, and 6 days postinfection. Mean viral titers ± SD are shown. Representative results are shown.

Mice were intranasally inoculated with 5×50% mouse lethal dose (MLD_50_) of influenza PR8 virus. In the first 2 days postinfection, no change in the general appearance of the mice was observed. However, reduced activity, ruffled fur, and difficulty breathing (tachypnea and labored respiration) were accompanied by reduced food and water intake at 3 days postinfection. From 4 days postinfection, these conditions, including weight loss (Figure 1B), diarrhea and cyanosis, worsened. At 6 days postinfection, half of the infected mice died. All of the remaining mice were dead by 9 days postinfection (Figure 1A). Viral loads were elevated in the lungs of the infected mice (Figure 1C).

### Macroscopic Findings of the Lungs and Pulmonary Edema

**Figure 2:**
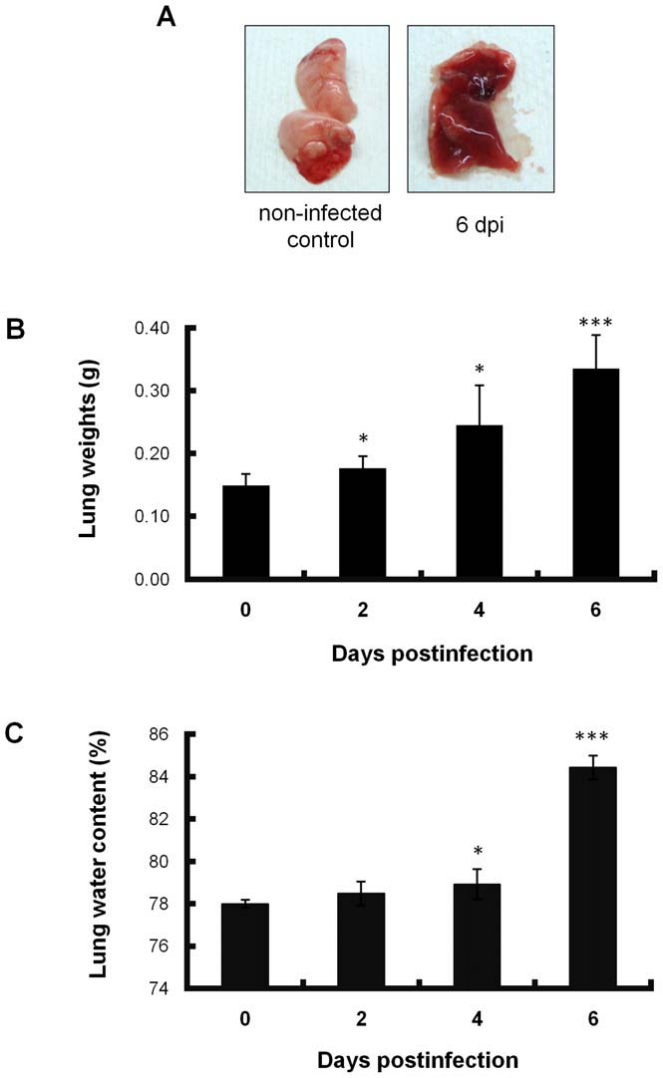
Macroscopic appearance, weight of lungs and water content in lungs of PR8-infected mice. (A) Gross pathology of mouse lung. Large, edematous and dusky red in color in infected lung at 6 days postinfection (right panel). Lung of non-infected control (left panel). dpi, days postinfection. (B) Weight of lungs excised from PR8-infected mice (n = 5, each day) sacrificed at indicated days. Significant differences were observed on days 2, 4 and 6 when compared with day 0 as a control. (C) Percentages of lung water content in PR8-infected mice (n = 5, each day) determined by dry-wet ratio assay modified by incubation at 50°C for 24 h. Significant differences were observed on days 4 and 6 when compared with day 0 as a control. Differences in means ± SD and *p* values are shown. *p<0.05, ***p<0.001. Data represent three independent experiments.

The lungs of infected mice were enlarged, edematous, and dusky red in color (Figure 2A). The weight of the lungs and the water content in the lungs gradually increased from 2 to 6 days postinfection (Figure 2B and C).

### Serial Histopathological Changes in the Infected Lungs

**Figure 3:**
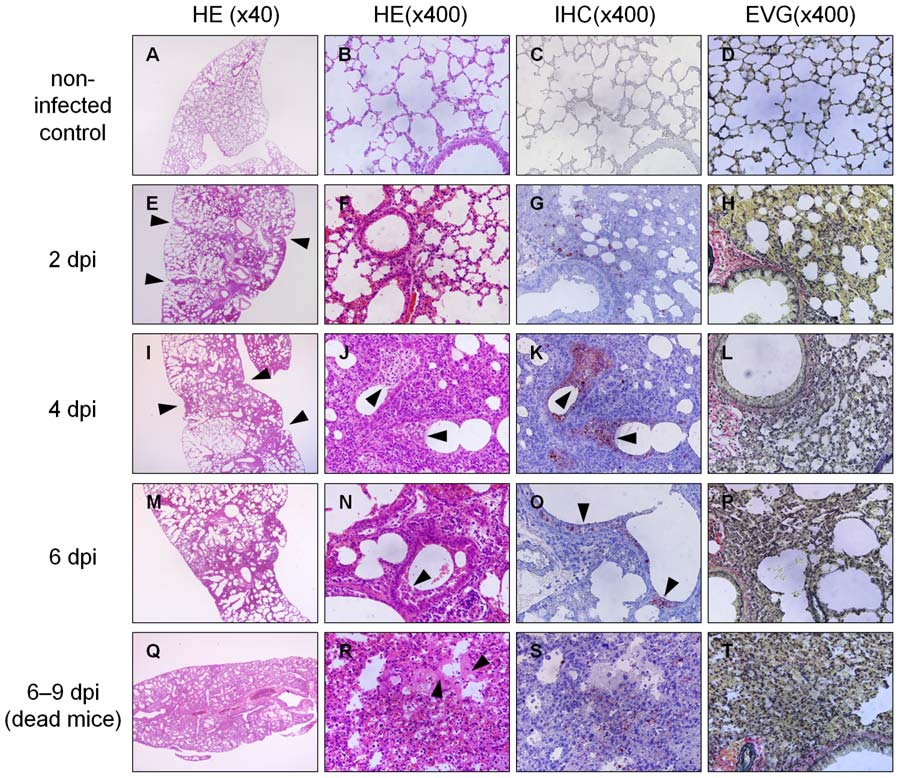
Serial histopathological changes in lungs infected with PR8 virus. (A–D) Non-infected mice were sacrificed and excised the lungs as a control. (E–P) Mice (n = 6, each day) were infected with PR8 influenza virus and sacrificed at the indicated days after infection. (Q–T) Dead infected mice were performed postmortem examination immediately after their death. (A, E, I, M and Q) Magnification is ×40. (B–D, F–H, J–L, N–P and R–T) Magnification is ×400. (A, B, E, F, I, J, M, N, Q and R) HE staining. (C, G, K, O and S) Immunohistochemical staining using anti-influenza virus polyclonal antibody. (D, H, L, P and T) elastica van Gieson (EVG) staining. (E–H) Infected lung at 2 days postinfection. (E) Focal inflammation in the lung and invagination of the pulmonary pleura (arrowheads). (F) Congestion in thick alveolar septa and mild collapse. (G) Antigen-positive cells are stained red in alveoli around bronchioles. (H) Mild alveolar collapse. (I–L) Infected lung at 4 days postinfection. (I) Expansion of inflammation and invagination of the pulmonary pleura (arrowheads). (J) Alveolar collapse and cell debris (arrowheads) in bronchiolar lumen. (K) Antigen-positive cell debris in bronchiolar lumen (arrowheads). (L) Increased alveolar collapse. (M–P) Infected lung from live mice at 6 days postinfection. (M) Lung deformation with enlargement of alveolar duct and alveolar collapse. (N) Cell debris in bronchiolar lumen (arrowhead). (O) Antigen-positive cell debris in bronchiolar lumen (arrowheads). (P) Alveolar collapse and enlargement of alveolar ducts. (Q–T) Infected lung from dead mice at 6 days postinfection. (Q) Severe collapse throughout entire lung. (R) Hyaline membrane formation lining alveolar ducts (arrowhead) and collapse. (S) Antigen-positive cells in collapsed area. (T) Severe alveolar collapse. HE, hematoxyline-eosin staining. IHC, immunohistochemistry. EVG, elastica van Gieson staining.

At 2 days postinfection, focal inflammation in alveolar septa, characterized by the infiltration of inflammatory cells, mononuclear cells and lymphocytes in the pulmonary parenchyma, was observed with mild alveolar collapse around a few bronchioles (Figure 3E, F and H). Invagination of the pulmonary pleura was caused by collapse (Figure 3E, arrowheads). The alveolar septa were thick and congested (Figure 3F). Infected cells were immunohistochemically stained positive in focal interstitial inflammation areas (Figure 3G), whereas other parenchyma, especially at the marginal area of the lung, were normal and infected cells were absent. The epithelium of bronchi and bronchioles was intact (Figure 3F). At 4 days postinfection, interstitial inflammation reached the distal parenchyma, and invagination of the pulmonary pleura expanded and became more severe (Figure 3I, arrowheads). Alveolar collapse and enlargement of alveolar duct were remarkable (Figure 3I, J and L). Infected cells were found in collapsed areas (Figure 3K). Cell debris that contained scrapped nuclei and stained positively with the anti-influenza antibody was observed prominently in the lumen of many bronchioles (Figure 3J and K, arrowheads), whereas the bronchiolar epithelium was normal. As shown in Figure 1A, there were both live and dead infected mice at 6 days postinfection. In the mice that were live at this point, infiltration with inflammatory cells, mainly mononuclear cells, lymphocytes and a few neutrophils, had further expanded throughout the lung (Figure 3M). Increasing alveolar collapse and enlargement of alveolar duct resulted in lung deformation (Figure 3M). The alveolar space beneath the pulmonary pleura contained proteinous exudates. Influenza antigen-positive cell debris accumulated in the lumen of bronchioles and antigen-positive cells in collapsed areas (Figure 3N and O, arrowheads). Only a few epithelial cells of bronchioles were infected. In contrast, in all of the dead mice from 6 to 9 days postinfection, DAD with hyaline membrane formation (Figure 3Q and R, arrowheads) and severe collapse (Figure 3Q, R and T) were broadly evident throughout the lungs in spite of the fact that several lungs were excised and fixed with paraformaldehyde immediately after their death. In addition, DAD with severe alveolar collapse were observed in dying but still live infected-mice at 8 days postinfection, which were sacrificed in accordance with a humane judgment because the mice should die within 24 hours. The collapsed areas contained several infected cells (Figure 3S). The distribution of pathological lesions, the degree of pulmonary edema, and the degree of DAD observed by microscopic examination are shown in Table S1. Histopathological assessment ruled out bacterial infection in all lungs.

### Hyaline Membrane Formation in the Infected Lungs

**Figure 4:**
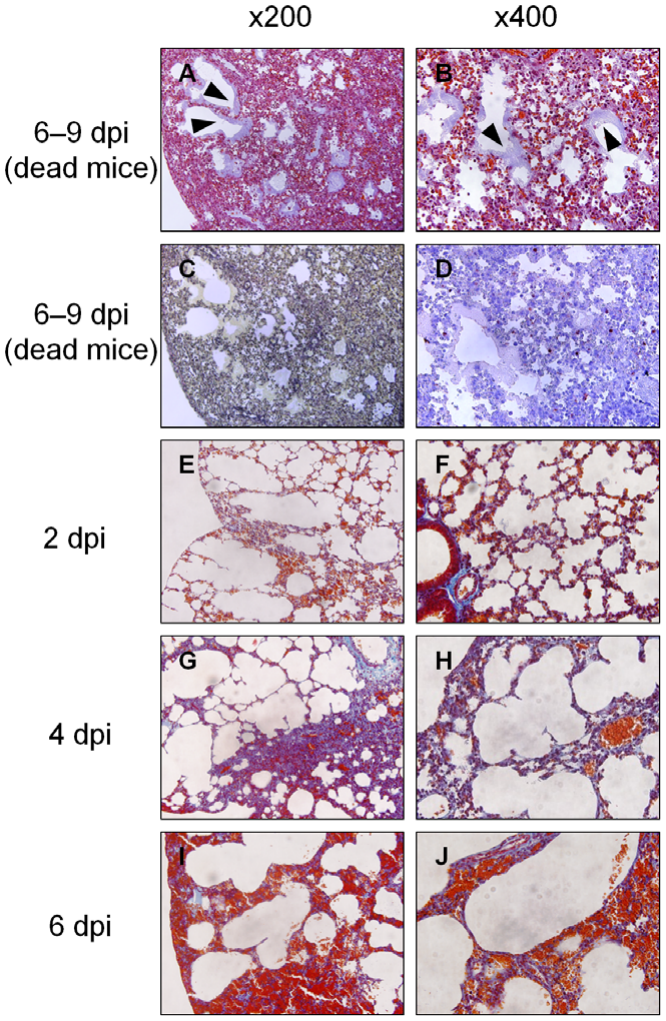
DAD with hyaline membrane formation demonstrated by Masson's trichrome staining. Postmortem examinations of dead infected mice were performed immediately after their death. (A and B) Hyaline membrane stained pastel purple (arrowheads) in enlarged alveolar ducts and alveolar spaces throughout collapsed lungs. (A) Magnification, ×200. (B) Magnification, ×400. (C) Elastica van Gieson staining shows alveolar collapse throughout entire lungs. Magnification, ×200 (D) Influenza virus antigen-positive cells in collapsed area beside hyaline membrane. Magnification, ×400 (E–J) Hyaline membrane was not found in any lungs from the live infected mice at 2–6 days postinfection. (E, G, and I) Magnification, ×200. (F, H, and J) Magnification, ×400.

Hyaline membrane formation was specifically visualized using Masson's trichrome staining. Pastel purple staining indicated that a thick hyaline membrane lined the alveolar ducts throughout the lungs (Figure 4A and B, arrowheads). Alveolar space was filled with hyaline membrane. Alveolar collapse was most evident around hyaline membrane formations (Figure 4C). Infected cells were located in collapsed parenchyma beside hyaline membrane (Figure 4D and 3S), but not in the hyaline membrane itself. In contrast, no hyaline membrane formation was observed in the lungs of the live infected-mice between 2 and 6 days postinfection (Figure 4E to J).

### Increase of Surfactant Protein (SP) -A and -D in Sera

**Figure 5:**
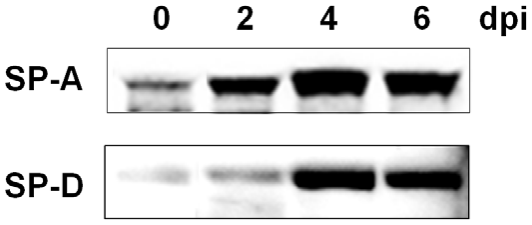
Levels of SP-A and -D in sera from PR8-infected mice. Upper and lower panels show SP-A and SP-D respectively, determined by Western blotting using the respective antibodies. Each lane was loaded with 10 µg of mouse serum without albumin and immunoglobulin.

Increased alveolar permeability and damage to the alveolocapillary interface in humans can be assessed by serum levels of SP-A and -D, which are markers of severe lung injury, interstitial pneumonia and acute respiratory distress syndrome [23]–[25]. Therefore, we examined SP-A and -D levels in sera from PR8-infected mice. The levels of both proteins were found to be increased between 2 and 6 days postinfection (Figure 5).

### Hyaline Membrane Formation with a Low Dose of Virus

**Figure 6:**
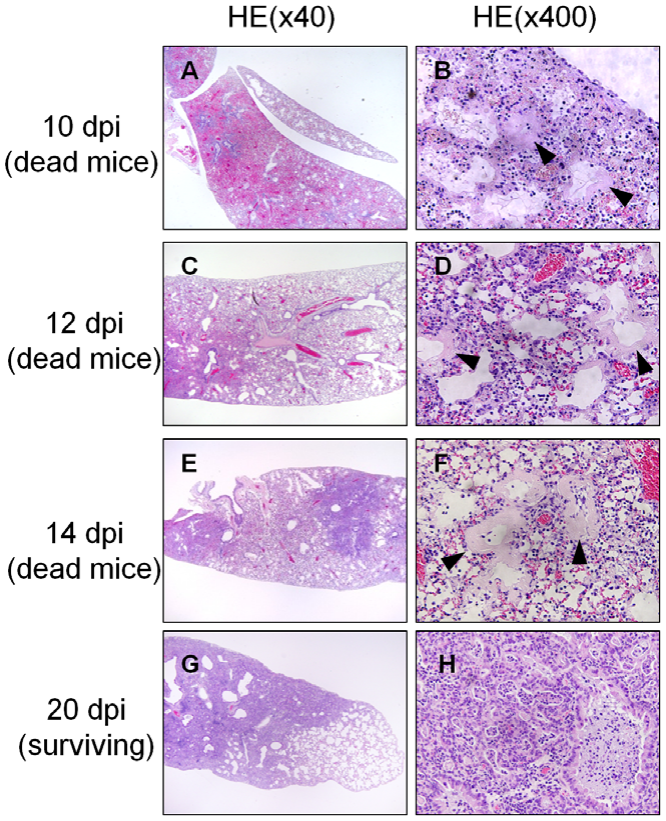
Hyaline Membrane Formation with a Low Dose of Virus. (A–F) Postmortem examinations of dead mice infected with 1× MLD_50_ of PR8 virus were performed at the indicated days. (A, C, and E) Magnification, ×40. (B, D, and F) Magnification, ×400. (A) Alveolar collapse and expanded alveolar ducts on 10 days postinfection. (B) Hyaline membrane lining alveolar ducts (arrowheads). (C) Alveolar collapse and expanded alveolar ducts at 12 days postinfection. (D) Hyaline membrane lining alveolar ducts (arrowheads). (E) Alveolar collapse and expanded alveolar ducts at 14 days postinfection. (F) Hyaline membrane lining alveolar ducts (arrowheads). (G and H) surviving mice infected with 1× MLD_50_ of PR8 virus were sacrificed at 20 days postinfection for histopathological examination. (F) Alveolar collapse, expanded alveolar ducts, glandular metaplasia, and thick alveolar septa were observed without hyaline membrane formation. Magnification, ×40. (G) Remarkable glandular metaplasia. Magnification, ×400.

To determine whether an inoculated virus dose affects hyaline membrane formation, we performed additional histopathological analysis of the lungs of mice inoculated with a low dose (1×MLD_50_) of PR8 virus. The infected mice died gradually between 9 and 16 days postinfection. In total, 50% of the infected mice survived for 20 days. Postmortem examination revealed hyaline membrane formation with collapse in alveoli and alveolar ducts in the dead mice, regardless of the number of days postinfection (Figure 6A to F, arrowhead). In contrast, when the surviving mice were sacrificed after the 20-day observation period, no hyaline membrane was observed anywhere in the lungs. Instead, glandular metaplasia, proliferation of bronchiolar epithelial cells extending to distal alveoli and alveolar ducts, with collapse and thick alveolar septa were broadly observed (Figure 6G and H).

## Discussion

The present study revealed the serial process of pathological changes from interstitial pneumonia to DAD in the lung of mice infected with influenza virus. Interstitial pneumonia was observed in the lung of live mice infected with PR8 virus when they were sacrificed. In contrast, DAD was found in the dying and dead mice. The histopathological characteristics of DAD, hyaline membrane formation, inflammatory cells accumulation and pulmonary edema, in the lungs of PR8-infected mice closely resembled the characteristics of DAD at the exudative stage in humans. Therefore, the serial pathological process from interstitial pneumonia to DAD observed in the PR8-infected mice is considered to be a model of the development of human DAD.

Our histopathological examination of live infected-mice sacrificed from 2 to 6 days postinfection revealed that interstitial pneumonia gradually expanded from pulmonary parenchyma around bronchioles to the entire lungs and DAD was not observed anywhere. All of the infected mice died from 6 to 9 days postinfection (Figure 1). DAD with severe collapse was found in the dying but still live mice at 8 days postinfection, which should die within 24 hours, and in all of the dead mice, several of which were autopsied immediately after their deaths (Figures 3 and 4). The results indicate that DAD is not a postmortem change. Considering histopathologically temporal alteration of the infected lungs, DAD with hyaline membrane appears to develop quickly, within at least 2 days. In clinical appearance, it is consistent with rapid progress to ARDS in human [11]. In addition, the dead mice inoculated with 1 × MLD_50_ of virus also developed DAD with collapse several days later than mice inoculated with 5 × MLD_50_ of virus (Figure 6). Moreover, in our preliminary study, when mice infected with 5 × MLD_50_ of PR8 virus were administered anti-influenza drug oseltamivir twice a day from 0 to 10 days postinfection, a few mice died after 12 days postinfection and the survival rate in the 20-day observation period was 80%. Histopathological examination of the live mice sacrificed on 8–10 days postinfection revealed that hyaline membrane with collapse was observed very focally in the lungs, in spite of the fact that the mice appeared not to die within at least 2 days. Taken together, these observations indicate that expansion of DAD in lungs is closely involved in mouse death in this model of influenza virus PR8 infection. This is compatible with the fact that DAD has been observed characteristically in autopsies of many patients who died of severe viral pneumonia and ARDS induced by influenza virus [5], [8], [26], [27].

The present study also revealed the order of hyaline membrane formation and alveolar collapse by continuous histopathological examination (Figure 3). Alveolar collapse in this study was observed earlier than previously reported [14]. Mild collapse and invagination of pulmonary pleura by alveolar collapse was already observed 2 days postinfection. Subsequently, collapse became more severe and significant expansion of alveolar duct was observed between 4 and 6 days postinfection as shown by hematoxylin-eosin (HE) and elastica van Gieson staining. In addition, the fact that invagination of pulmonary pleura became more severe and wider as shown in low magnification of HE staining is implicated in augmentation of alveolar collapse. Finally the expanded alveolar ducts were lined by hyaline membrane in the dying and dead mice. As described above, hyaline membrane formation followed severe alveolar collapse in this study. Katzenstain have mentioned that alveolar collapse is found in the fibrotic stage of DAD [14], but at least in mouse infected with influenza virus, alveolar collapse was found earlier than hyaline membrane formation in the exudate stage of DAD. Several events involved in alveolar collapse were also observed in the present study. After 4 days postinfection, antigen–positive cell debris was apparently increased in bronchioles, suggesting that pulmonary cells were destroyed by influenza virus infection downstream from the bronchioles. Destruction of the pulmonary cells indicates the disruption of the alveolar-capillary barrier. In fact, surfactant proteins, SP-A and SP-D, drastically increased in the serum and pulmonary edema occurred in infected mice (Figures 5 and 2). Disruption of the alveolar-capillary barrier also induces an influx of serum content into alveolar space. Moreover, the water and protein contents in flowing serum dilute lung surfactant and inhibit its function. In addition, decreases in the quantity of type II pneumocytes by virus infection may be directly linked to the absence of lung surfactant because type II pneumocytes produce surfactant. Based on the fact that lung surfactant prevents alveolar collapse [28], qualitative and quantitative loss of lung surfactant may result in alveolar collapse in mouse lungs infected with influenza virus. Lung surfactant has also been shown to reduce surface tension at the alveolar air–liquid interface and stabilize alveoli and terminal airways at low lung volumes [23]. Therefore, decreases in lung surfactant in the infected lung may lead to the malfunction the alveolar–capillary barrier, e.g., the further augmentation of influx and disruption of efflux of protein-rich exudate which originated in serum, and eventually the formation of hyaline membrane. According to this perspective, the widespread alveolar collapse observed in the infected lungs might imply the formation of hyaline membrane.

Immunohistochemical examination in this study revealed influenza virus antigen–positive cells in pulmonary parenchyma around bronchioles (Figures 3 and 4). Interestingly, only a few epithelial cells of bronchioles were infected with influenza virus and their structure remained intact throughout the observation period. This finding indicates that influenza virus infection to bronchiolar epithelium is not necessary for the development of DAD. Furthermore, hyaline membrane formation was observed following the appearance of infected necrotic debris within bronchioles, suggesting that the destruction of alveoli, rather than bronchioles, is a key to the development of DAD with hyaline membrane, which corresponds with previous findings [14].

In conclusion, the present study demonstrated the pathological process from interstitial pneumonia to DAD with severe collapse in mice infected lethally with influenza A virus H1N1. Furthermore, expansion of DAD in the lungs was found to be correlated with mouse death in influenza virus infection. These findings indicate that, in addition to anti-influenza virus drugs, inhibition of the development of DAD with alveolar collapse by medical intervention may relieve severe pneumonia and ARDS by influenza virus and improve the survival rates.

## Materials and Methods

### Mice and Virus

Female 5- to 6-week-old, specific pathogen-free BALB/c mice verified as being uncontaminated with pneumonia-causing pathogens, such as *Pasteurella pneumotropica*, *Mycoplasma pulmonis,* and Sendai virus, were obtained from Japan SLC, Inc. (Hamamatsu, Japan). Mouse-adapted PR8 virus, influenza A/Puerto Rico/8/34 (A/PR/8/34, H1N1), was kindly provided by the National Institute of Infectious Diseases, Japan [29] and was grown once in the lung of BALB/c mouse. The homogenate of the infected lungs were clarified by low speed centrifugation at 3,500 rpm for 5 minutes at 4°C, and the supernatant were used as purified virus. The PR8 virus titer were measured by plaque forming assay using Madin-Darby canine kidney (MDCK) cells.

### Cells and Antibodies

MDCK cells were obtained from the American Type Culture Collection (ATCC CCL-34). The cells were maintained in minimum essential medium (MEM) containing 10% fetal bovine serum, 50 units/ml penicillin, and 50 µg/ml streptomycin. The cells were cultured in 5% CO_2_ at 37°C. Rabbit anti-human influenza A, B virus polyclonal antibody (code #M149, Takara, Tokyo, Japan) was used for immunohistochemical analyses. Rabbit anti-SP-A antibody (Santa Cruz Biotechnology, Inc., Santa Cruz, CA) and mouse anti-SP-D monoclonal antibody (Abcam Inc., Cambridge, MA) were used for Western blot analysis of mouse blood samples purified using Aurum serum protein mini kits (Bio-Rad, Hercules, CA).

### Viral Infection of Mice

The mice were anesthetized with isoflurane and were intranasally inoculated with 1× or 5×50% mouse lethal dose (MLD_50_) of PR8 virus for infection studies. Mice (n = 8) were inoculated with 1×MLD_50_ of virus for survival rate, clinical condition and body weight. Mice (n = 40) were also inoculated with 1×MLD_50_ of virus and 3 of them were sacrificed every two days for lung histopathological analysis. Mice (n = 10) inoculated with 5×MLD_50_ of virus were monitored for survival rate, clinical condition and weighed daily. Mice (n = 54) inoculated with 5×MLD_50_ of virus were sacrificed for lung histopathological analysis. Mice (n = 60) were inoculated with 5×MLD_50_ of virus and 5 of them were sacrificed at 0, 2, 4, and 6 days postinfection to measure infectious viral loads in the lungs, lung weight and lung water content. Dead mice were also dissected for lung histopathology. The amount of water in the infected lungs was measured using a modified dry-wet ratio assay [30]. Briefly, the lungs were excised from the PR8-inoculated mice and weighed (wet weight). After incubation at 50°C for 24 hours, the lungs were again weighed (dry weight). The amount of water in the lungs was determined by subtracting the dry weight from the wet weight. The protocols of all mouse experiments were approved by the Animal Care and Use Committee of the National Center for Global Health and Medicine of Japan (approval ID: 21-I-9).

### Plaque Forming Assay

Plaque forming assays were performed to measure virus loads in the lung of infected mice as previously described [31]. In brief, the infected mice were sacrificed at 0, 2, 4, 6 days postinfection. Their lungs were excised, homogenated using Multi-beads Shocker (Yasui Kikai Co., Osaka, Japan), and clarified by centrifugation at 3,500 rpm for 5 minutes at 4°C. The clarified supernatants containing virus were diluted serially using MEM containing 0.2% bovine albumin, 2 mM L-Glutamine, 50 units/ml penicillin, and 50 µg/ml streptomycin. The diluted viruses were infected to an MDCK monolayer for 1 hour at 37°C. The MDCK monolayer was washed with PBS (−) once to remove free viruses, and overlaid with MEM containing 0.6% agar, and incubated at 37°C. After incubation for 2 days, the monolayer cells were stained with crystal violet solution (0.095% crystal violet and 19% methanol).

### Histopathological Analysis

Mouse lungs were fixed in 4% paraformaldehyde and stained with hematoxylin and eosin (HE). Elastica van Gieson method [32] was performed to visualize easily crumpling alveoli without air space because elastic fiber in alveolar septa was stained black. Masson's trichrome staining [33] was performed to visualize hyaline membrane formation. Immunohistochemical analysis was performed to detect influenza virus antigens. In brief, the histopathological slides were deparaffinized and treated with methanol containing 3% hydrogen peroxide to block endogenous peroxidase. The slides were incubated with rabbit anti-human influenza A, B virus polyclonal antibody (code #M149) [34] and were treated with HistoMouse Max (Invitrogen, San Diego, CA) in accordance with the manufacturer's instructions [35]. Histopathological evaluation was measured by microscope investigation. The slides were viewed using an Olympus BX51 microscope and software DP controller to capture images.

### Statistical Analysis

All data are expressed as means ± SD. Differences between groups were assessed using Student's *t* test, and *p*<0.05 was considered statistically significant.

## Supporting Information

Table S1 **Results of observation of the lungs from mice infected with 5× MLD_50_ of PR8 virus by the microscopic examination.**dpi, days postinfection. focal/diffuse, the affected area was focal or diffuse, it depended on samples. (−), not observed. (±), faint. (+), obvious. (++), severe.
(TIF)
