# Supplementary figures and images for: Serial Histopathological Examination of the Lungs of Mice Infected with Influenza A Virus PR8 Strain
Source: PLoS One. 2011 Jun 20;6(6):e21207. doi: 10.1371/journal.pone.0021207 (PMC3118813; doi:10.1371/journal.pone.0021207)

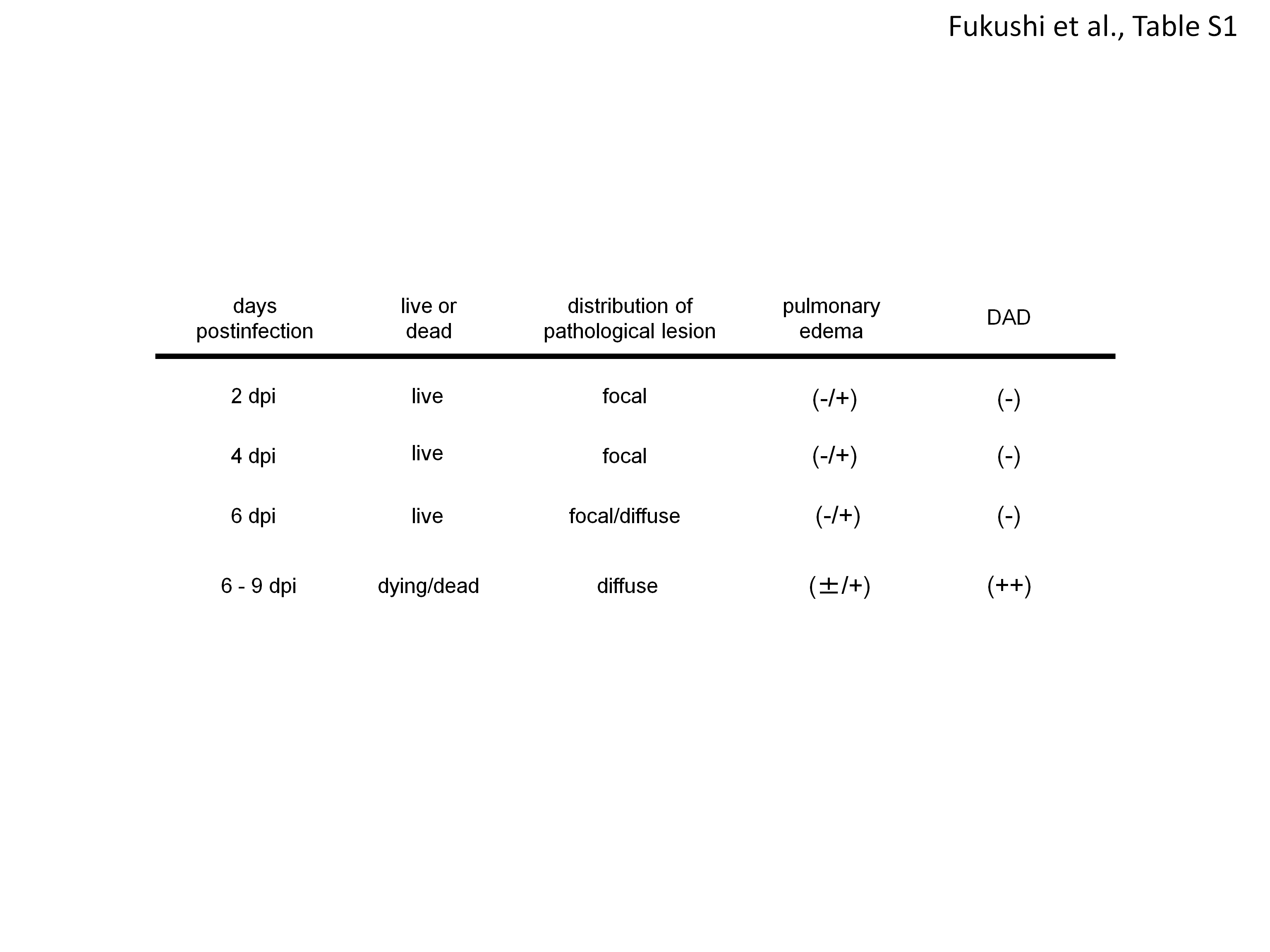

Supplement: Table S1 — Results of observation of the lungs from mice infected with 5× MLD50 of PR8 virus by the microscopic examination.dpi, days postinfection. focal/diffuse, the affected area was focal or diffuse, it depended on samples. (−), not observed. (±), faint. (+), obvious. (++), severe. (TIF) [file pone.0021207.s001.tif]
